# Extensive Summary of the Important Roles of Indole Propionic Acid, a Gut Microbial Metabolite in Host Health and Disease

**DOI:** 10.3390/nu15010151

**Published:** 2022-12-28

**Authors:** Hui Jiang, Congying Chen, Jun Gao

**Affiliations:** State Key Laboratory of Pig Genetic Improvement and Production Technology, Jiangxi Agricultural University, Nanchang 330045, China

**Keywords:** indole propionic acid, gut microbiota, gut–organ axis, gut barrier, tryptophan

## Abstract

Increasing evidence suggests that metabolites produced by the gut microbiota play a crucial role in host–microbe interactions. Dietary tryptophan ingested by the host enters the gut, where indole-like metabolites such as indole propionic acid (IPA) are produced under deamination by commensal bacteria. Here, we summarize the IPA-producing bacteria, dietary patterns on IPA content, and functional roles of IPA in various diseases. IPA can not only stimulate the expression of tight junction (TJ) proteins to enhance gut barrier function and inhibit the penetration of toxic factors, but also modulate the immune system to exert anti-inflammatory and antioxidant effects to synergistically regulate body physiology. Moreover, IPA can act on target organs through blood circulation to form the gut–organ axis, which helps maintain systemic homeostasis. IPA shows great potential for the diagnosis and treatment of various clinical diseases, such as NAFLD, Alzheimer’s disease, and breast cancer. However, the therapeutic effect of IPA depends on dose, target organ, or time. In future studies, further work should be performed to explore the effects and mechanisms of IPA on host health and disease to further improve the existing treatment program.

## 1. Introduction

The diversity and homeostasis of gut microbial communities play an important role in host health and nutrient metabolism [1]. More and more evidence suggests that metabolites produced by gut microbiota are key mediators of the cross-talk between dietary intake and host health [2,3]. Animal cells cannot synthesize tryptophan [4]. Although some gut bacteria, such as *Escherichia coli*, can produce tryptophan, the contribution of bacterial-derived tryptophan to the physiological functions of the body has not yet been reported [4]. Therefore, as an essential amino acid, tryptophan is mainly taken up by humans from the diet [5], especially from protein-rich foods, such as meat, eggs, milk, and chocolate. Among the 20 most common amino acids, tryptophan is the most complex and the one with the least content in cells and proteins, but it plays an indispensable role in body metabolism [6].

As an essential macronutrient, proteins are used by humans to meet the requirements of the body [7]. Most dietary proteins are digested and absorbed in the upper gastrointestinal (GI) tract by the action of proteases. According to the varied intake, some proteins, peptides, and amino acids can enter the large intestine following the peristalsis of the gut tract [8]. Finally, amino acids in the gut tract are deaminated or decarboxylated by microbiota to form various small-molecule metabolites [9]. The efficiency of protein fermentation in the distal intestine tract is higher than that in the proximal section [10,11]. Similarly, phenolic compounds, which are bacterial metabolites of aromatic amino acids, are at least four times more abundant in the human distal colon than in the proximal colon [12]. This is likely because the ability of bacteria to digest protein is strengthened by the consumption of carbohydrates, the extended digestion time, the higher pH values [13], and the increase in the number of bacteria [14].

With the increasing interest in the interaction between host health and small-molecule microbial metabolites, studies are emerging consecutively on metabolites generated by microbiota, such as short-chain fatty acids (SCFAs) [15] and secondary bile acids [16], which mediate the interaction between the host and the microbial community and have made outstanding contributions to human health and disease treatment. Moreover, progress has been made in the technical aspects of physical and mental health intervention and treatment for the host through dietary intervention [17] and fecal microbial transplantation (FMT) [18]. In addition to metabolites from the metabolism of carbohydrates [19], bacterial metabolites from protein metabolisms also play an important role in host physiology [20]. Previous results have shown that approximately 5% of dietary tryptophan is metabolized by gut microbiota [21], and the resulting tryptophan metabolites are important signaling molecules in the microbial community, acting as a bridge for host–microbe interaction, which is essential for the maintenance of the gut micro-ecosystem homeostasis [22]. In particular, indole propionic acid (IPA), as a small-molecule metabolite produced only by microbial degradation [23], has been increasingly explored for its role in host health. For instance, a 1-year follow-up of preselected impaired glucose tolerance (IGT) participants in the Finnish Diabetes Prevention Study (DPS) reported that participants without diabetes had a mean serum IPA level of 1.095 μM, compared to 0.894 μM in participants with diabetes [24]. The concentration of IPA in the serum of healthy adults measured by Alexeev et al. was approximately 50 nM [25], while the IPA levels in the plasma of healthy adults and the systemic blood of rats were 1.011 μM [26] and 5.079 μM [27], respectively. Additionally, according to the study of Pavlova et al., the content of methyl-IPA in the urine of pregnant women was 0.522 μM [28]. The concentration of IPA in rat feces is approximately 10 μM, although that in the human gut has not been clearly reported [27]. Although the concentration of IPA in adults varies in different reports, the order of metabolites of tryptophan from high to low concentrations is as follows: indole, indole acetic acid (IAA), and IPA [13]. Despite the lack of research, IPA has emerged for treating various diseases in models (e.g., breast cancer and NAFLD) [29,30], and is expected to play a role in treating more diseases in the future.

Here, we review recent progress in the studies of IPA and discuss the investigation into its potential roles in mediating microbe–host interactions. Given that existing studies linking tryptophan metabolites to health have often been obtained based on in vitro cell cultures or mouse-related models, more research is needed to provide information on the relationship between IPA and human health.

## 2. IPA, a Metabolite of Tryptophan Metabolism by Gut Microbiota

In the early 1980s, researchers hypothesized that IPA was produced by gut microbiota and could pass through the blood–brain barrier as a result of its decreased concentration in the cerebrospinal fluid of mice after taking antibiotics [31]. IPA could be detected in the plasma of conventional (conv) mice, but not in mice that had microbial colonization for less than 5 days [32]. Additionally, high levels of serum IPA by intraperitoneal injection are rapidly cleared from the blood within a short time in GF animals, indicating that serum IPA levels in animals are dependent on commensal gut microbiota.

The bacterial strains that produce IPA are mainly identified by isolation and in vitro culture [33]. Elsden et al. found that *Clostridium sporogenes*, *Clostridium botulinum*, and *Clostridium caloritolerans* can produce IPA in tryptophan metabolism. Jellet et al. also identified IPA in *Clostridium cylindrosporum* medium, except for *C. sporogenes* [34] (Table 1). Additionally, Biancone et al. conducted in vitro culture experiments on 16 gut bacteria from six orders and identified that *Peptostreptococcus asaccharolyticus* could produce IPA [12]. Importantly, different culture conditions interfere with the identification of IPA-producing bacteria. For example, environmental factors, such as low pH value, significantly inhibit the ability of microbiota to metabolize tryptophan to produce IPA [12].

**Figure 1:**
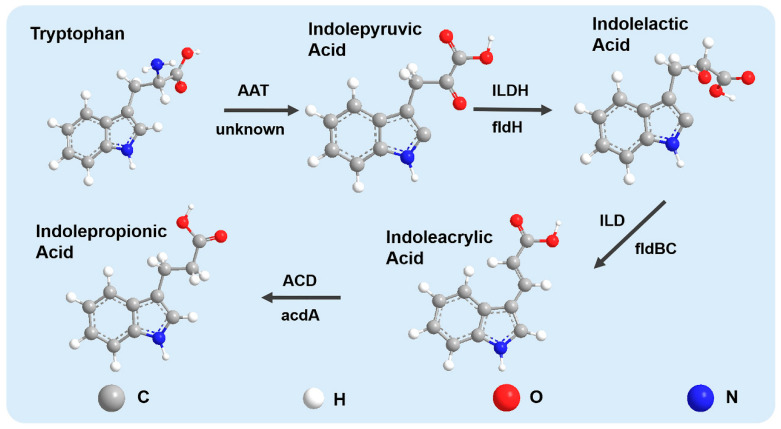
Metabolic process of dietary tryptophan converted to IPA by gut microbiota. The active enzymes required for this process are indicated above the arrow, and the genes encoding these enzymes in *C. sporogenes* are shown below. The enzymatic activity of AAT has been demonstrated in *C. sporogenes* cells, but so far, the gene encoding this enzyme has not been identified. AAT: Aromatic amino acid aminotransferase, ACD: Acyl-CoA dehydrogenase, ILD: Indolelactate dehydratase, ILDH: Indolelactate dehydrogenase. The ball-and-stick models of the molecules involved in the figure were drawn using ChemDraw (https://www.chemdraw.com.cn/ (accessed on 16 June 2022)) and chem3D (https://www.wavemetrics.com/project/Chem3D (accessed on 16 June 2022)).

Until now, the pathway producing IPA has been mostly investigated in *C. sporogenes*. IPA is universally known to be produced from tryptophan via the reductive pathway. Based on the biological process of *C. sporogenes* decomposing phenylalanine to produce phenylpropionic acid, Dodd et al. predicted that the process of metabolizing tryptophan to produce IPA is similar in genetics, and they subsequently identified the gene involved in IPA production (Figure 1). By knocking out the fldC gene, the ability to produce IPA was lost [35]. Further identification experiments in in vitro medium showed that the presence of the fldBC gene cluster was a reliable marker for the production of IPA, which provided an important reference for the subsequent preliminary screening of IPA-producing bacteria.

Simultaneously, Wlodarska et al. discovered a complete fldAIBC gene cluster in *Peptostreptococcus russellii* and *Peptostreptococcus anaerobius* genomes through genome sequencing. This cluster imparts that ability to metabolize tryptophan into IPA, while *Peptostreptococcus stomatis* can still synthesize a small amount of IPA despite the lack of the activator fldI [36].

In addition to diet and gut microbiota, many human studies have suggested that the ACSM2A gene is an important factor affecting circulating levels of IPA by genome-wide association analyses [23,37,38]. Given that ACSMs are involved in the glycine coupling pathway and metabolize/detoxify organic acid metabolites, polyphenols produced by gut microbiota, and long-chain fatty acids (MCFAs) [39], Menni et al. suggested that ACSM2A may be involved in the metabolism and excretion of IPA, rather than related to its production [37].

So far, the discovery of IPA-producing bacteria has relied on in vitro experiments, which limits the identification of more relevant bacteria. The integrated analysis of gut microbiota, metagenome, and IPA metabolism should facilitate the detection of more IPA-producing bacteria in future studies.

## 3. The Effect of Different Dietary Patterns on IPA Production

IPA is a metabolite of dietary tryptophan produced by gut microbiota [40]. Thus, changes in gut microbial composition, as well as dietary patterns, can affect IPA production.

Menni et al. found that IPA was positively correlated with the α-diversity of gut microbiota, and gut microbiome composition could explain approximately 20% of the variation in cycling levels of IPA [37]. Several studies have found that circulating levels of IPA correlate with fiber intake [24,41], which may be explained by the changes in gut microbiota. In addition, the polyphenol-rich diet led to a significant increase in serum IPA in subjects with normal renal function, but not in subjects with impaired renal function [42]. Consistent with this, the β-diversity of the microbiome composition in the cecum and colon of pigs fed inulin for 60 days was increased significantly, accompanied by a significant increase in IPA content [43].

Of course, different dietary structures can also change the level of IPA. For example, Mediterranean diet led to an increase in IPA levels after the treatment for only 4 days, while fast food (i.e., fries and burgers) induced the opposite result [44]. In addition, plasma IPA levels were significantly higher in the normal diet-fed littermates than in the ketogenic diet-fed mice [45]. A study in 117 overweight adults found that the intake of fried meat reduced the richness of the gut microbial community and led to a decrease in IPA concentrations [46]. Pimentel et al. explored the effects of fermented dairy products on the human serum metabolome through a randomized crossover study design in 14 healthy men [47]. Compared with the milk group, the postprandial blood concentrations of IPA and IAA in the yoghurt intake group were lower. Similarly, healthy overweight men with mildly elevated C-reactive protein levels were given a 500 mL postprandial shake (consisting of 300 mL custard, 150 mL cream cheese, and 50 mL whipping cream) after receiving the Anti-Inflammatory Dietary Mix (AIDM) (consisting of a range of dietary products with anti-inflammatory properties) in a postprandial challenge trial [48]. AIDM subjects exhibited reduced plasma concentrations of indole-3-propionic acid in the late stage.

In summary, we hypothesized that different dietary structures alter the composition of gut microbes, which leads to individual variability in IPA production. Unfortunately, there is a lack of clear experimental validation for the effect of dietary components, such as fiber, on IPA production.

## 4. The Roles of IPA in Host Diseases and Health

**Figure 2:**
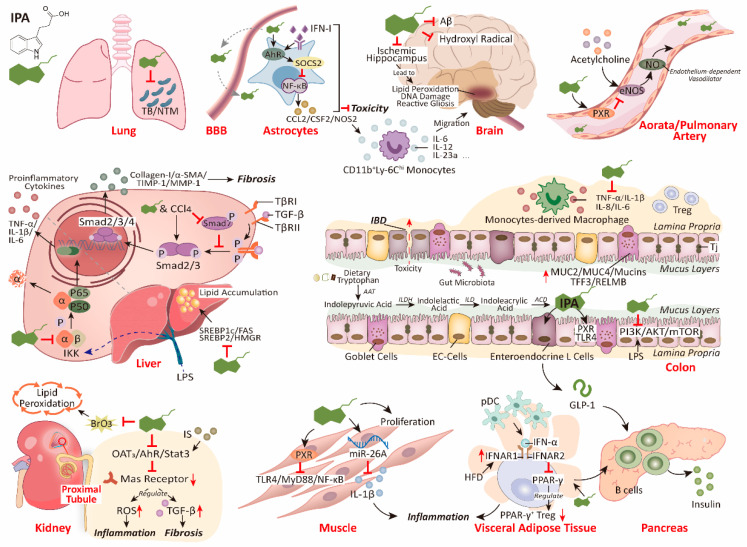
Biological functions of IPA through various gut–organ axes. Blood circulation enables IPA to act on various target organs, regulating host disease and health through biological mechanisms as shown. Aβ: Amyloid β-protein, BBB: Blood–brain barrier, eNOS: endothelial nitric oxide synthase, GLP-1: Glucagon-like peptide-1, HFD: High-fat diet, IBD: Inflammatory bowel disease, IFN-I: Type I interferons, IS: Indoxyl sulfate, LPS: Lipopolysaccharide, NTM: Non-*Mycobacterium tuberculosis*, PXR: Pregnane X receptor, ROS: Reactive oxygen species, TJ: Tight junction, TB: Tuberculosis.

Dietary tryptophan can be catabolized to IPA by gut microbiota in the colon. Based on previous studies, IPA has important biological functions in many diseases. Critically, compared to IAA, IPA has an increased ability to cross the blood–brain barrier, transport to target organs with blood circulation, activate corresponding receptors, and participate in the life activities of the host. Therefore, IPA may affect body health through the gut–brain axis, gut–liver axis, gut–lung axis, and other pathways (Figure 2). Therefore, understanding the production of microbial metabolites and the signal pathways through which they affect host health may provide new ideas and schemes for disease treatment. Here, we reviewed the mechanism of IPA in various metabolic diseases and immune homeostasis, and provided novel ideas for disease prevention and mitigation.

### 4.1. IPA Protects the Brain from Disease and Oxidative Damage

As the most abundant glial cells in the central nervous system (CNS), astrocytes can not only support and enhance the survival of neurons [49], but they also respond to inflammatory signals, promote inflammatory responses, and participate in the regulation of multiple physiological processes of the nervous system [49,50]. The important functions of astrocytes have led researchers to study their many roles. Indeed, Sanmarco et al. found that astrocytes can induce T-cell apoptosis to reduce CNS inflammation and specified the regulatory role of gut microbes in this pathway [51]. Nuclear translocation of NF-κB is a critical step in the functioning of astrocytes, which promotes various pathological processes including experimental autoimmune encephalomyelitis (EAE). This step is regulated by various pathways, which can be roughly divided into the drivers and inhibitors of NF-κB activation [50]. Using the EAE mouse model, Rothhammer et al. found that type I interferons (IFN-Is) induced aryl hydrocarbon receptor (AHR) expression in astrocytes, which activated the suppressor of cytokine signaling 2 (SOCS2), interfered with NF-κB activation, and prevented NF-κB from binding to Ccl2, Csf2, and Nos2 promoter [52]. Ultimately, this process inhibits the recruitment of inflammatory monocytes to the CNS, controlling the pathogenic activity of astrocytes during EAE. Consistent with other results, in this process, various AHR agonists, including IPA, which are produced through metabolizing tryptophan by gut microbes [53], are involved in the negative regulation of IFN-I-induced inflammation by reducing IL-6, IL-12, and other inflammatory factors. These results indicate that microbial metabolites play a vital role in the regulation of the nervous system and immune function.

Both amyloid beta-peptide deposition [54] and mitochondrial damage [55] contribute to the development of Alzheimer’s disease (AD). With the deepening of the understanding of the gut microbiome, numerous studies have attempted to explain the underlying mechanism of AD from the perspective of gut microbiota [56,57], and to improve cognitive status through probiotics [58] or FMT [59]. Studies have found that microbial metabolites, such as trimethylamine N-oxide (TMAO) [60], lipopolysaccharide (LPS), and SCFAs [61], are associated with amyloid β-protein (Aβ) deposition in the brain. As reported previously, Aβ-induced neuronal damage and dysfunction are often associated with reactive oxygen species (ROS) [62,63]. Many studies have found that melatonin has neuroprotective properties [64,65], although the clinical efficacy is suboptimal [66]. IPA has a heterocyclic aromatic ring structure similar to that of melatonin. It is an effective hydroxyl radical scavenger and can effectively protect nerve cells from oxidative damage by Aβ (1–42) [67]. Hydroxyl radicals have both strong activity and toxicity, and cannot be detoxified by enzymes. Antioxidants have evolved into endogenous scavengers in the early stages of life evolution, thus providing on-site protection against oxidative damage [68,69]. IPA is an endogenous electron donor that detoxifies highly reactive free radicals by donating electrons to hydroxyl anions, and kynuric acid is the end product of hydroxyl radical-mediated oxidation of IPA [68]. Importantly, IPA does not undergo side chain decarboxylation like IAA to generate reactive peroxyl radicals, so no pro-oxidative intermediates are generated [70]. The synergistic effect of IPA and glutathione have been shown to effectively inhibit the formation of 2,2′-azino-bis-(3-ethyl-benz-thiazoline-6-sulfonic acid) (ABTS) cationic free radicals mediated by hydroxyl radicals [71]. Additionally, IPA has receptor-mediated mitochondrial protection function in nerve cells by enhancing the mitochondrial respiration rate, increasing membrane potential, and reducing the production of ROS to inhibit the occurrence of AD [72]. Furthermore, IPA produced by normal metabolism has no cytotoxicity and can exist stably. This phenomenon means that levels of oxidative damage to proteins may increase due to decreased levels of antioxidants, such as IPA. However, the pathogenesis of Alzheimer’s disease is complex. In addition to the extracellular aggregation of Aβ plaques, intracellular aggregation of neurofibrillary tangles (NFTs) caused by excessive phosphorylation of τ- protein is also an important pathological feature [73]. Moreover, the aggregation of Aβ plaques is widely distributed in critical stages. Whether IPA has a promising application for the treatment of AD requires more in-depth experimental investigation in conjunction with pathogenesis in the future.

Liu et al. found that an intermittent diet can increase serum IPA content and alleviate diabetes-induced cognitive impairment [74]. Additionally, IPA can prevent lipid peroxidation and DNA damage in the hippocampus after transient forebrain ischemia, and inhibit gliosis, thereby protecting neurons from ischemic injury [75]. These findings may be related to the antioxidant and mitochondrial protective properties of IPA. Interestingly, in Huntington’s disease (HD), another neurodegenerative disorder caused by the amplification of N-terminal repeats in the huntingtin protein, the IPA content in the plasma is significantly decreased [26] and may represent an effective marker of HD. Moreover, supplementation with probiotics helps to inhibit the depressive behavior of Sprague–Dawley (SD) rats and upregulate the content of IPA in the plasma [76]. It is gratifying that the positive role of IPA in neurological diseases is becoming increasingly recognized by researchers. Indeed, IPA has been used in the clinical development of the neurological disorder, Friedrich’s ataxia [77]. Thus, IPA has become a research hotspot in the research and clinical usage of the gut–brain axis.

In conclusion, some indole compounds, such as IAA and indole-3-pyruvic acid, show antioxidant activity. However, they are also accompanied by the formation of reactive intermediates and result in lipid peroxidation [70,78]. These accompanying metabolites bring risks to the treatment of related diseases. IPA produced by microbiota can pass the blood–brain barrier into the cerebrospinal fluid, target nerve cells, and participate in the regulation of brain activity and the secretion of inflammatory factors. Although the current research is mainly limited to cell culture in vitro or animal models, these studies have still provided an attractive approach for treating human diseases.

### 4.2. IPA Inhibits Liver Fibrosis and Lipotoxicity by Reducing Inflammation

Nutrients and other metabolites in the gut pass through the portal vein and are transferred to the liver, which forms an “axis” (i.e., the gut–liver axis), through which the gut and liver can interact. Accordingly, all types of gut factors may affect the biological metabolic processes of the liver and regulate their physiological functions [79]. The dysbiosis of gut microbiota produces a series of toxic substances that enter the enterohepatic circulation through the damaged gut barrier and aggravate the progress of liver diseases. The integrity of the gut barrier is beneficial for delaying or preventing the occurrence and development of many diseases [80].

Non-alcoholic fatty liver disease (NAFLD) is a metabolic syndrome related to hepatic manifestation, which mainly includes non-alcoholic fatty liver (NAFL), non-alcoholic steatohepatitis (NASH), NASH-related cirrhosis, and hepatocellular carcinoma (HCC) [81]. Globally, the prevalence of NAFLD-related HCC has been increasing following the incidence of obesity and has become a major public health problem endangering human health. Altered gut microbial structure [82] and decreased immune surveillance [83] both lead to NAFLD-related HCC development and require further investigation. To date, there is still a lack of reliable drug regimens for treating NASH [84]. Therefore, it is crucial to constantly explore potential treatment strategies for NASH.

Zhao et al. alleviated hepatic steatosis and damage, and restored metabolic homeostasis in high-fat diet (HFD) rats, by oral administration of IPA over 8 weeks [29]. Specifically, IPA treatment significantly reduced the levels of plasma alanine transaminase (ALT), aspartate aminotransferase (AST), liver triglyceride, cholesterol, and the degree of infiltration of neutrophils and macrophages. IPA also downregulated the expression of genes related to liver fibrosis and collagen synthesis and inhibited the pathological process of liver disease. Briefly, the biological mechanism of this process is that IPA treatment significantly inhibits the phosphorylation of p65, IκBα, and IKKα/β in the upstream signaling pathway of NF-κB [85], thereby reducing the expression of its downstream targets of inflammatory factors (TNFα, IL-1β, and IL-6) and chemokine (CCL2 and CCR2) expression. In vitro hepatic macrophage exposure experiments also demonstrated that IPA directly dose-dependently inhibited LPS-induced activation of the NF-κB signaling pathway. Additionally, oral administration of IPA can reduce the ratio of Firmicutes and Bacteroidetes increased by HFD, reshape the structure of gut microbiota, upregulate the expression level of TJ proteins, reduce gut epithelial permeability, and inhibit the production of endotoxin leakage.

Zhang et al. found that high-fat/high-cholesterol (HFHC)-fed mice developed cholesterol-related dysbiosis, impaired microbial tryptophan metabolism, and significantly decreased serum IPA levels compared to high-fat/low-cholesterol (HFLC) [86]. In vitro experiments have shown that IPA could inhibit cholesterol-induced lipid accumulation and cell proliferation. In addition to the upregulated expression of pro-inflammatory cytokines in the serum and liver, high cholesterol can induce oxidative stress and activate hepatic stellate cells, which promote liver fibrosis [87] and the development of NAFLD-HCC. More importantly, HFHC can also lead to increased serum LPS concentrations in the portal vein of mice and the loss of colonic E-cadherin, suggesting that excess cholesterol impairs gut barrier function, and the leaky gut further exacerbates the severity of the disease [88].

Hyperlipidemia is a global epidemic. Various evidence has indicated that the incidence of hyperlipidemia is sex-biased [89,90]. Sex-dependent treatment strategies and drugs are urgently required. Numerous studies have shown that AHR is a key factor in regulating lipid metabolism [91], and the activation of AHR negatively regulates various adipogenesis genes, such as SREBP1c and FAS [92]. Indole derivatives, such as IAA, indoxyl sulfate (IS), and IPA, play various physiological functions by binding to AHR [4,21,93]. Li et al. found that IPA can dose-dependently reduce the transcription of key genes for fatty acid and cholesterol biosynthesis in the liver. This indicates that IPA may act as an AHR ligand to mediate the sex-differentiated hypolipidemic effect of 1-deoxynojirimycin (DNJ) through the IPA-AHR lipid metabolism axis [94], but the mechanism remains to be determined.

However, the regulation of IPA on the physiological functions of the liver is not always positive and beneficial. Recently, it has been reported that the addition of IPA can exacerbate the expression of hepatic inflammatory factors caused by CCl_4_, thereby activating the transforming growth factor-β1 (TGF-β1) signaling pathway and inducing an increase in phosphorylation levels of Smad2/3 [95]. This undoubtedly enhances the activation of the hepatic stellate cells and eventually leads to excessive deposition of extracellular matrix (ECM) [96]. In the Smads signaling pathway, Smad7 has been identified as a key inhibitor of liver fibrosis, while Smad2/Smad3 functions as a promoter [97]. Notably, oral IPA alone did not cause liver damage and fibrosis, nor did it affect liver malondialdehyde (MDA; an indicator of lipid peroxidation) [98] and antioxidant levels. Similarly, Sehgal et al. also found that circulating IPA was significantly lower in patients with liver fibrosis, especially those without type 2 diabetes mellitus (T2DM), compared to individuals without fibrosis [99]. IPA exerts the potential to protect the liver by inhibiting cell migration and cell adhesion of the human hepatic stellate cell line (LX-2), which are hallmark features of hepatic stellate cell (HSC) activation.

This is consistent with the findings of Liu et al. that IPA has different effects on oxidative stress under different conditions [95]. Studies have shown that IPA depends on PXR and AHR receptors to enhance the oxidative and nitrosative stress of breast cancer cells, reduce the proportion of cancer stem cells, enhance anti-tumor immunity, and ultimately inhibit the proliferation and metastasis of cancer cells to improve the survival rate of patients [30]. Additionally, higher concentrations of IPA can inhibit Fe^3+^- [100] and Cr^3+^-induced [101] oxidative damage; both metal ions can induce cancer via the Fenton reaction.

In the liver, IPA can directly inhibit the activity of the NF-κB signaling pathway and the production of pro-inflammatory cytokines. IPA also inhibits NF-κB by upregulating the expression of TJ proteins, restoring the gut barrier, and preventing TLR4 activation by enteric endotoxin. The TLR4/NF-κB signaling pathway is also involved in the formation of liver fibrosis. The inhibition of the hepatic NF-κB signaling pathway by IPA may explain the remission of liver fibrosis [102]. However, IPA can aggravate CCl_4_-induced liver fibrosis through the TGF-β1 signaling pathway in mouse models, indicating that IPA may interact with some substances in vivo to produce adverse effects, which will become a restrictive factor in IPA treatment [95]. Cytochrome P450 enzymes (CYPs) in the liver are involved in cholesterol synthesis and metabolism [103], and PXR induces the expression of hepatic CYPs [104]. CYP7A1 is a key rate-limiting enzyme in the breakdown of cholesterol into bile acids [105]. PXR has been shown to be activated by pregnenolone 16α-carbonitrile (PCN) and mediated its inhibition of CYP7A1 expression, suggesting that potent PXR agonists may be an effective scheme for treating cholestasis [106,107]. CCl_4_ is also metabolized by CYPs in the liver [108]. However, contrary to the findings described above, serum total bile acid levels were significantly elevated after IPA supplementation. These studies suggest that IPA might affect bile acid metabolism by stimulating CYPs through PXR and interacting with CCl_4_. 

At present, many studies have proved that IPA can inhibit lipid accumulation and reduce the levels of triglycerides, cholesterol [29], and low-density lipoprotein cholesterol (LDL-c) [94] in the liver. However, whether it acts through AHR and a specific metabolic pathway needs to be verified. Based on the close association between IPA and lipid metabolism, subsequent studies may attempt to explore the interaction between IPA and obesity-related metabolic phenotypes by association analysis. The effect of IPA on rodents under basic conditions and the minimum effective and safe dose for treating NASH will need to be determined for the clinical application of IPA in the future.

### 4.3. IPA Inhibits Endogenous or Exogenous Substance-Induced Kidney Injury

Changes in the gut microbiota lead to the production of uremic toxins, which play an important role in the development and progression of chronic kidney disease (CKD). Growing evidence has linked IS/PCS (p-cresol sulfate) to kidney diseases, such as glomerulosclerosis [109,110], while IPA may be an important biomarker for preventing the development of CKD and as a kidney protector [111].

As a uremic toxin, IS is absorbed by proximal tubular cells (HK-2) through OAT1 and OAT3 on the membrane, and downregulates the expression of Mas receptors through the OAT3/AHR/Stat3 pathway [112]. Inhibition of Mas receptors increases ROS production, which activates the NF-κB pathway [113] and stimulates the expression and activity of TGF-β1 [114,115]. These physiological changes lead to renal dysfunction, such as renal interstitial fibrosis and inflammation, and accelerate the progression of CKD, especially in HK-2. Shimizu et al. also demonstrated that transcription 3 (Stat3) is involved in the expression of genes related to IS-induced fibrosis and inflammatory gene expression in HK-2 cells [116]. Surprisingly, IPA, which is also a metabolite of dietary tryptophan, inhibits Stat3 activation, downregulates the expression of IS-induced fibrosis genes (TGF-β1) and inflammatory factors (monocyte chemoattractant protein-1), and protects the host kidney from damage [117].

IPA also has potential protective effects against diseases induced by exogenous toxic substances. For example, KBrO_3_ is a carcinogen that can cause oxidative stress [118]. Although it was banned from the food processing industry, the presence of KBrO_3_ in the environment has still harmed human health [119], largely via targeting the kidney and thyroid to induce tumorigenesis [120]. Classic antioxidant enzymes, such as superoxide dismutase (SOD) and catalase, have little protective effect on KBrO_3_-induced oxidative damage. However, IPA can significantly inhibit KBrO_3_-induced renal and serum lipid peroxidation and protect the kidney from damage [121]. Of course, IPA treatment is also effective against thyroid lipid peroxidation in rats due to KBrO_3_ injection [122]. It is worth mentioning that IPA is a poor chain-breaking antioxidant. Indeed, KBrO_3_-induced oxidative stress in porcine thyroid homogenate is not effectively inhibited by IPA in vitro, and higher concentrations are required to reduce endogenous MDA formation in rat striatal homogenate in vitro [71].

Taken together with previous studies, patients with CKD are often accompanied by attenuated antioxidant defenses and increased oxidative stress [123], and oxidative stress is significantly associated with increased kidney damage. The protective effect of serum IPA on the development of CKD can be explained by its strong antioxidant ability because IPA is an effective hydroxyl radical scavenger and does not produce toxic effects under basic conditions. Additionally, IPA is an organic anion that can compete with some toxic substances, such as IS, for organic anion transporters (OATs) and reduce its accumulation in the proximal renal tubules. Reducing renal injury may be a protective mechanism of IPA in the kidneys [124]. By measuring the content and ratio of IPA and IS in healthy and diseased individuals, a threshold range is established to predict the progress of CKD, which is expected to provide a new direction for the invasive diagnosis of diseases.

### 4.4. IPA Protects the Lungs from Bacterial and Fungal Infections

Tuberculosis (TB) is a traditional disease caused by *Mycobacterium tuberculosis*, which can cause damage to multiple organ systems. TB is mainly a lung disease [125] that can be transmitted through the air and represents a serious threat to global human health [126]. Therefore, this pathogen plays an important role in disease progression, and the high prevalence of drug-resistant *M. tuberculosis* strains is a pressing medical issue [127]. The development of efficient and stable anti-tuberculosis drugs is essential to reduce TB mortality. More recently, Dumas et al. reported that microbiota contribute to early host resistance to pulmonary colonization of *M. tuberculosis*. Antibiotic mice are more susceptible to *M. tuberculosis* infection compared to the control group [128]. 

The key enzymes in the important metabolic pathways of *M. tuberculosis* are targets for developing novel anti-tuberculosis drugs. The aromatic amino acid biosynthesis pathway is essential for the survival of *M. tuberculosis* [129], so the key enzymes in this pathway become potential targets for developing new anti-TB drugs. Anthranilate synthase (AS) catalyzes the first step in tryptophan biosynthesis, which is the synthesis of anthranilate from glutamine and chorismate. This step can be feedback-inhibited by tryptophan [130]. The AS complex contains two functional domains named AS component I (ASI) and II (ASII), which are encoded by the genes trpE and trpG, respectively [131,132]. Recent studies have shown that IPA can block tryptophan biosynthesis and exert its antibacterial activity by mimicking Trp as an allosteric inhibitor of ASI in the tryptophan synthesis pathway of *M. tuberculosis* [133]. Negatu et al. further demonstrated the resistance and pharmacokinetic properties of IPA in a mouse model of TB, forecasting exciting new advances in the field of microbial prevention and treatment of infectious diseases [134]. Importantly, after IPA treatment, the bacterial load in the lungs of mice infected with *M. tuberculosis* aerosol decreased without adverse reactions. IPA not only shows antibacterial activity against some clinically resistant *M. tuberculosis* and non-*Mycobacterium tuberculosis* (NTM) species, but also induces programmed cell death of *Candida albicans* dependent on Ca^2+^ [135] (Table 2). However, under experimental conditions, IPA has no antibacterial activity against some Gram-positive bacteria (*Staphylococcus aureus*) and Gram-negative bacteria (*Escherichia coli*, *Pseudomonas aeruginosa*, and *Acinetobacter baumannii*) [133,134]. At present, IPA appears to show broad-spectrum anti-mycobacterial activity.

Early in vitro experiments found that IPA is also a potent inhibitor of the growth of *Legionella pneumophila*, and the bacteriostatic effect is enhanced with increased IPA concentration and exposure time. Thus, IPA inhibits pulmonary inflammation caused by bacteria [136]. *Legionella pneumophila* has a higher incidence of pneumonia, which is more likely to develop into severe community-acquired pneumonia (SCAP) than other atypical respiratory pathogens, which even necessitates patients to be admitted to the intensive care unit (ICU) [137]. Interestingly, tryptophan supplementation reduced the efficacy of IPA. We speculate that this could be explained by the findings of Negatu et al., who showed that IPA binds to TrpE to inhibit the tryptophan synthesis pathway, and tryptophan supplementation in vitro alleviates this inhibitory effect to some extent.

HIV-positive patients are generally more susceptible to TB infection [138], an important factor in increased mortality, and co-infection of the two diseases can complicate treatment by the interaction between anti-retroviral and anti-tuberculosis drugs [139,140]. Notably, IPA may be a potential pharmaceutical ingredient in such treatments. Some studies have found that the content of IPA decreased significantly in patients with HIV treated with anti-retroviral therapy (ART) [141], which is an important marker to distinguish HIV infection from healthy people [142]. The mechanism of this effect is still unclear, but it can be partly explained by the impaired gut barrier leading to the translocation of LPS and LPS-binding protein (LBP), which aggravates the systemic inflammatory response. Moreover, patients with HIV treated with ART are also prone to NAFLD/NASH [143,144], further illustrating the multiple therapeutic potentials of IPA in various diseases.

Comparatively, IPA is a small-molecule metabolite with good pharmacokinetic properties, which can be easily absorbed by the host to play a full role in therapy. In the future, IPA is expected to improve the existing treatment options for some diseases through its potential as a complement to anti-tuberculosis and anti-retroviral drugs. It is worth mentioning that, except for *M. tuberculosis*, the inhibitory effect of IPA on other bacteria and fungi has not been verified in vivo, which will become an important research subject in the future.

### 4.5. IPA Promotes Muscle Growth and Relieves Muscle Inflammation

Sarcopenia, first named in 1988, is an age-related progressive skeletal muscle disease and a chronic muscle inflammation that occurs mostly in elderly adults [145]. The clinical symptoms of sarcopenia include loss of muscle mass, functional decline, susceptibility to falls, and even disability [146]. Owing to the close connection between muscles and bones, it is common for patients with sarcopenia to develop osteoporosis [147]. If not treated in time, sarcopenia will further lead to impairment of mobility, reduced quality of life, and increased treatment burden.

Muscle is the largest storehouse of protein in the body, and an imbalance in protein catabolism can lead to excessive protein degradation, muscle loss, and dysfunction [148]. The composition of gut microbiota and its metabolites are closely related to the host phenotype, and supplementation with probiotics [149], prebiotics [150], or fecal transplantation [151] can effectively interfere with the development of sarcopenia. Some microbial metabolites, such as LPS [152], SCFAs [153], and IS [154], can affect the production, metabolism, and quality of muscle to different degrees. Importantly, various nutrients and metabolites produced by gut microbiota can reach and act on muscles, so intervention of the gut–muscle axis may be a new target for modulating muscle function [155].

Recent studies have shown that the colonization of *C. sporogenes* increases the IPA content, regulates the expression of myogenic regulatory factors, and effectively promotes the increase in muscle fiber diameter and muscle cross-sectional area of quadriceps in experimental mice [156]. Previous studies have reported that PXR reduces the secretion of inflammatory factors and alleviates inflammatory diseases by inhibiting the NF-κB signaling pathway [157,158]. In addition to activating PXR receptors in muscle cells, IPA inhibits the TLR4/MyD88/NF-κB signaling pathway by inducing miR-26A expression, which ultimately downregulates the expression of pro-inflammatory markers (CCL2, CCL5, IL-1β, and TNFα). However, the causal relationship between the activation of PXR and the overexpression of miR-26A has not yet been elucidated, both of which are important for reducing muscle inflammation. Meanwhile, IPA-induced overexpression of miR-26A specifically targets the 3′UTR region of IL-1β mRNA, inhibiting its transcription and reducing inflammation. Consistently, *Clostridium XIVa* may help to relieve sarcopenia [159], and the species of *Clostridium XIVa* are potential candidates for the production of IPA [94]. However, whether the species of *Clostridium XIVa* can produce IPA needs further verification.

Although the current research on the effects of IPA on muscle function is relatively rare, its remarkable effects on reducing inflammation, promoting muscle growth, and improving muscle have attracted increasing attention. Combining the current knowledge related to muscle inflammation and gut microbial metabolites with biomarker research, nutritional intervention, and drug development is crucial to promote the clinical practice and development of early disease prevention, diagnosis, and treatment.

### 4.6. IPA Has the Potential to Safeguard Insulin Secretion to Prevent T2DM

T2DM is a metabolic symptom characterized by hyperglycemia and insulin resistance [160]. As morbidity continues to rise globally, particularly in lower-income countries, effective public health and clinical interventions are an effective way to reduce the global healthcare burden [161]. Age, genetics, lifestyle, and obesity play key roles in the pathologic progression of T2DM [162]. Moreover, the GI tract represents an important target for diseases as it is the largest immune organ in the body [163]. FMT has achieved promising results for treating T2DM, emphasizing the important role of gut microbiota and their metabolites in T2DM [164,165]. Ley et al. found that compared to control mice, obese mice had a higher ratio of Firmicutes/Bacteroidetes (F/B) in the gut [166]. A similar result was obtained in a human study [167]. Many studies have found that the F/B ratio is elevated in patients with T2DM [168,169]. Moreover, IPA can significantly reduce the HFD-induced increase in F/B ratio [29]. Similarly, Konopelski et al. found that IPA supplementation inhibited weight gain in mice [27]. Jennis et al. found that the IPA content decreased significantly in obese patients with T2DM, which was significantly reversed 3 months after Roux-en-Y gastric bypass surgery (RYGB) [170].

Dietary intervention is another important method for T2DM treatment [171]. Indeed, IPA-rich diets can reduce the blood glucose concentration and homeostatic model assessment (HOMA) index in SD rats [172], which is consistent with the results by Menni et al. [37]. However, IPA supplementation failed to modulate depression-related behavior in rats, contradicting previous findings [76]. A previous DPS showed that serum concentrations of IPA and several lipid metabolites can be used as markers for identifying T2DM, considering that high serum IPA could protect patients from diabetes [41]. Surprisingly, the IPA content is positively correlated with the intake of dietary fiber and carbohydrate, but there are few reports to suggest that IPA can be obtained from fiber fermentation [24,37]. The latest study also verified the reliability of this result, and also found that except for *Bifidobacterium*, all IPA-related bacterial genera were associated with fiber intake in the same direction as the association between IPA and bacterial genera [23]. One possible reason for this is that fiber intake increases the number of fiber-degrading bacteria [173], some of which can produce IPA or its substrates from tryptophan [13]. We found that the hypothesis is well supported by previous reports indicating that diets containing fiber and low-fat, high-complex carbohydrates have a protective effect on T2DM [174]. *Bifidobacterium* is associated with the variant in the LCT locus that determines lactose tolerance [175] and is also identified as a novel locus of IPA in GWAS analysis [23]. Compared with lactose-tolerant individuals, lactose-intolerant individuals had higher *Bifidobacterium* and IPA. Although the evidence that *Bifidobacterium* can produce IPA is insufficient [12], many studies have found that some strains of *Bifidobacterium* can produce ILA, an IPA substrate [176,177]. ILA can be converted to IPA acid by gut microbiota. And when ILA is below the optimal level, IPA production will be affected [178]. Therefore, in lactose-intolerant individuals, *Bifidobacterium* has more lactose as an energy source to promote its reproduction [179], which facilitates the generation of IPA.

The colon has the highest density of enteroendocrine L cells, and the microbial metabolite indole can stimulate the secretion of glucagon-like peptide-1 (GLP-1) from enteroendocrine L cells [180]. Hence, Mello et al. proposed that IPA may also stimulate enteroendocrine L cells to secrete GLP-1 [41]. The gut-derived peptide GLP-1 can act on β cells through the gut–insulin axis to promote insulin secretion [181]. GLP-1 has been reported to suppress appetite, control energy intake, and enhance satiety through the gut–brain axis, which may reduce the risk of obesity-induced T2DM and play a key role in the pathogenesis of T2DM [182,183]. Tuomainen et al. reported that IPA tends to be associated with insulin secretion and is significantly negatively correlated with serum high-sensitivity C-reactive protein (hsCRP) levels in their follow-up study on DPS [24]. They also proposed that the potential benefit of IPA in reducing the risk of T2DM may be inseparable from its reduction in inflammation and protection of β cells. Peroxisome proliferator-activated receptors (PPARs), especially α and γ, play an important role in the regulation of glucose and lipid homeostasis, and AHR agonists can increase the expression of PPAR-α [184]. Surprisingly, Kuhn et al. proposed that IPA with a specific structure can act as PPARα/γ co-agonists and has the potential to treat T2DM and dyslipidemia [185]. PPARγ is associated with insulin sensitivity and is the target of a drug approved for treating T2DM [186,187,188]. Whether IPA can inhibit the progress of T2DM, and whether IPA plays a regulatory role alone or through activating AHR, still needs reliable research verification. Of course, the protective effect of IPA on T2DM is closely related to its strong antioxidant stress ability, which may play a role in protecting β cells from oxidative stress-related damage and guarantee insulin secretion.

In conclusion, IPA is extremely important in improving gut microbial composition and relieving T2DM, opening a new horizon for T2DM treatment. Based on the existing research results, dietary fiber can promote the production of GLP-1 [189,190], and fiber-degrading bacteria that are significantly related to IPA may also play a synergistic role in this process. Given the complexity of T2DM, there are still many unknown mechanisms between IPA and T2DM that need to be elucidated. It is impossible to determine whether IPA and gut microbes co-stimulate GLP-1 secretion alone or synergistically, and we cannot exclude the possibility that IPA directly acts on pancreatic islets. Therefore, further studies are needed to elucidate the mechanism of IPA in regulating the enteroendocrine system and metabolic homeostasis, including glucose metabolism.

### 4.7. Differential Regulation of Cardiovascular Function by IPA in Different Receptors and Time Contexts

Cardiovascular disease (CVD) is a complex disease that is affected by many factors, including diet, age, genetics, and lifestyle [191,192,193]. Atherosclerosis is the major potential risk factor for CVD [194]. CVD, including hypertension, cerebrovascular disease, and coronary heart disease, is related to metabolism and seriously threatens human health [195]. For a long time, researchers have used multi-omics technology to investigate the pathogenesis of CVD [196,197,198] and work on drug development to better treat diseases and reduce the medical burden of patients [199,200]. Therefore, it is of great biomedical significance to identify potential targets for the prevention and treatment of cardiovascular diseases. Gut microbial composition [201] and its metabolites [202] are closely related to cardiovascular function, and they have become a key factor for regulating human cardiovascular diseases, resulting in some significant achievements [203].

Gesper et al. identified IPA as a regulator of mitochondrial respiration in murine cardiomyocytes (HL-1) [204]. Specifically, chronic exposure (24 h) to IPA induced mitochondrial dysfunction in HL-1 following stimulation with the uncoupler carbonyl cyanide-4-(trifluoromethoxy) phenylhydrazone (FCCP), which was also observed in the human hepatoma cell line (Huh7) and human umbilical vein endothelial cells (HUVECs). Consistent with acute treatment (30 min), which increased mitochondrial maximal respiration in HL-1, IPA dose-dependently enhanced cardiac contractility in an off-topic mouse cardiac perfusion model. Note that the above experimental phenomena are all produced under cellular stress conditions (FCCP) and IPA fails to alter the basal respiration of HL-1. Overall, acute IPA treatment is beneficial for CVD, but follow-up experiments should explore the potential pitfalls of long-term IPA administration and its mechanism of action.

Previous studies have compared patients with advanced atherosclerosis with the sex- and age-matched control group, and found that the content of baseline plasma IPA decreased significantly [205]. They also found that IPA was significantly positively correlated with the ankle-brachial index (ABI), reflecting the blood supply status of the lower extremities. IPA was also significantly negatively correlated with arterial stiffness in the Twins UK cohort comprising female twins [37]. However, paradoxically, indoles normally act as ligands for the AHR. The activation of AHR induces macrophage activation and foam cell formation in apolipoprotein E (ApoE) knockout mice, participates in vascular inflammation, and eventually develops atherosclerosis [206]. Additionally, Lee et al. recently found that IPA fails to treat Western diet (WD)-induced atherosclerosis, and high circulating levels of IPA may be harmful [21]. In view of the variability in the results of studies on atherosclerosis, we propose several following hypotheses. First, regarding differences in the composition of gut microbiota, IPA in drinking water was also ingested as a food component during the experiment, and prolonged exposure to a WD gradually shapes the gut microbial composition, making it difficult to recover lost microbial species even with the introduction of dietary fiber [207]. Eventually, different gut microbiota compositions lead to different changes in host phenotypes [173]. *Bifidobacterium* is negatively correlated with vascular function impairment [208]; HD decreases *Bifidobacterium* [209], and its quantities in the SD group were also significantly reduced by IPA supplementation [21]. Second, regarding the mode of administration and dosage of IPA, there is currently no clear report on the effective dose of IPA, while oral gavage is known to be less affected by the environment. Third, the absorptive and metabolic profiles of the experimental populations are different, particularly differences in substance absorption capacity and basic physical conditions between mice fed a normal diet and WD [210]. Fourth, the circulating IPA concentration in the SD + IPA group was twice compared to that in the WD + IPA group, both of which were significantly higher than those in the control group. In addition, the higher circulating levels of IPA led to metabolic dysfunction [21]. Moreover, overexpression of AHR, which occurs in mice supplemented with IPA, masks the benefits of IPA, and many studies have reported that AHR overexpression can lead to malignant phenotypes such as cancer [211,212]. Finally, IPA works synergistically with other metabolites, and the benefits of IPA in atherosclerosis may require other metabolites, with the desired effect being difficult to achieve when IPA is used alone. All the above hypotheses need to be further confirmed.

Huc et al. reported that a tryptophan-rich diet increased blood IPA levels and portal blood pressure (PBP) in rats compared to a tryptophan-free diet [213]. Although IPA inhibits CNS inflammation by stimulating AHR [52], the modulation of cardiovascular function by IPA may be mediated by different receptors. PXR acts as an endogenous ligand and is widely distributed throughout multiple tissues, including the gut, liver, and breast [214,215]. Pulakazhi et al. found that IPA inhibited the release of endothelial nitric oxide synthase (eNOS)-dependent NO by activating the vascular endothelial PXR receptor and ultimately reduced agonist-induced endothelium-dependent vasodilation (such as in the aorta and pulmonary artery) [216]. The specific biological mechanism by which PXR regulates eNOS expression has not yet been clarified. Toell et al. reported that the presence of two GGTTCA motifs is directly repeated and is separated by a distance of four nucleotides (DR4) in the promoter region of the inducible nitric oxide synthase (iNOS) gene in the DLD-1 human epithelioid-like colorectal adenocarcinoma cells to form the response element (RE) of PXR [217]. Heterodimerization between PXR and retinoid X receptor (RXR) on DR4-type RE mediates clotrimazole-induced upregulation of iNOS mRNA. Therefore, Lee et al. speculated that IPA may be involved in the negative regulation of PXR-RXR REs, downregulating eNOS, and leading to eNOS upregulation in PXR^−/−^ mice [21]. Of course, further research is needed to verify this hypothesis. Admittedly, the regulation mechanism of PXR itself on blood vessels is also very complex. As described by Hagedorn et al., progesterone metabolites such as 5β-dihydroprogesterone act on mesenteric arterial PXR receptors in mice and enhance the cytochrome P450 epoxygenase activity, helping to regulate vasodilation to accommodate pregnancy [104]. Based on the existing studies, the mechanisms of vascular function regulation are diverse, and they are mainly related to tissue type (mesenteric arteries or aorta), agonist type (IPA or 5β-dihydroprogesterone), receptor type (AHR or PXR), and targeted enzyme type (eNOS or cytochrome P450 epoxygenase).

Cardiovascular disease represents a significant problem in humans. Given the complex and diverse mechanisms regulating vasoconstriction or vasodilation, it will be challenging to explain the role of IPA in blood pressure regulation and cardiovascular disease prevention and treatment, although it still has significance for drug development in clinical treatment. We introduced the current research progress on IPA regulation of the vascular state and listed the differences and possible mechanisms of different studies to provide ideas for further in-depth research.

## 5. Protection of IPA on the Gut Barrier

The GI tract provides a habitat for numerous microbiota, and accumulating evidence suggests that the gut microbiota are important in supporting the epithelial barrier [218,219]. The gut barrier is mainly divided into epithelial and mucous barriers, and it consists of a layer of epithelial cells connected by TJ proteins. Many factors, including mucin, active molecules, and immune factors, work together to maintain the integrity of this barrier [220]. However, when these factors are abnormal, gut permeability may increase, resulting in a leaky gut. The well-functioning gut–organ axis relies on integrated gut barriers and healthy gut microbiota structure. Therefore, modulating the interaction of the gut microbiota and the gut barrier could serve as a novel strategy for treating some gut and extra-gut diseases.

Studies have found that IPA is closely associated with higher microbial diversity [37], and probiotic supplementation increases the content of IPA and may mediate some immunomodulatory effects [76]. We divided the protection of IPA on the GI tract into two aspects: barrier maintenance and immune metabolism. Jennis et al. found that IPA dose-dependently reduced the permeability of T84 cells induced by interferon-γ (IFN-γ) and tumor necrosis factor-α (TNF-α) [170]. Recent studies have shown that IPA enhances the viability of HT-29/CACO-2 cells, promotes the expression of TJs (claudin-1, occludin, and ZO-1), reduces paracellular permeability, and improves gut barrier function against LPS-induced damage [221]. In addition, IPA can enhance the gut mucus barrier, such as by increasing goblet cell secreted products (TFF3 and RELMβ) and mucin production (MUC2, MUC4), and improving goblet cell function [36,221]. This is supported by the study of Wlodarska et al., who also found that IPA supplementation increased MUC2 expression in a co-culture system of bone marrow-derived macrophages and colon spheroids [36]. Studies have shown that the gut mucus layer of patients with IBD is thinner and the glycosylation of mucin 2 (MUC2) is reduced [222,223]. These changes may reduce the adaptability of commensal microorganisms, causing microbial disturbances. As expected, the number of bacteria using α-L-fucosidase to cut terminal fucose residues in mucin is significantly reduced in patients with UC and CD [36]. In addition, the phenomenon of fewer *fldAIBC* clusters in patients with IBD supports the benefits of IPA in the mucus barrier. Fortunately, not only in mice but also in human patients with active UC, the content of IPA is significantly decreased, and its content recovers with the remission of the disease [25]. Based on the above, we conclude that mucin provides sugar substrates for the colonization of tryptophan-metabolizing bacteria, and microbial metabolites such as IPA can ensure the normal secretion function of goblet cells, thus forming a benign and stable circulatory system to jointly ensure the homeostasis of the gut environment.

In clinical diseases, paraquat (PQ) poisoning usually causes mucosal damage and has a high mortality [224]. Yu et al. found that compared to the control group, the IPA content in the mouse model with acute PQ poisoning was significantly reduced, which was accompanied by a decrease in the abundance of IPA-producing bacteria *C. botulinum* and *P. anaerobius* [225]. This suggests that the oxidative damage caused by PQ can be partly attributed to the reduction of gut bacteria with the function of producing antioxidant metabolites resulting in damage to the gut mucosa. Ultimately, the loss of effective gut protection increases the systemic distribution of toxins. This hypothesis is supported by the potential negative correlation between IPA and LPS (markers of gut microbiome translocation) in the plasma of HIV-infected patients, as mentioned above [141]. These results indirectly suggest that IPA can regulate gut permeability and prevent toxin leakage. As Yusufu et al. demonstrated, a tryptophan-deficient diet not only resulted in gut micro-ecological imbalance but also systemic inflammation in elderly mice [226]. IPA-producing gut microbes, PXR expression in epithelial cells, and the presence of TLR4 are essential for maintaining a normal gut barrier and function in multiple mouse models of gut inflammation [227]. Loss of IPA or PXR or overexpression of TLR4 will result in impairment of the gut barrier, which undoubtedly leads to toxin leakage and systemic spread. As a member of the nuclear receptor superfamily, PXR is considered a new drug target for inflammatory bowel disease (IBD) [228].

The gut barrier is inextricably linked to the state of immune activation [229], and gut microbiota influence host immunity through various metabolites, including products of microbial tryptophan metabolism such as IPA. For example, mice colonized with the fldC mutant of *C. sporogenes* do not normally secrete IPA [35]. Compared to normal mice, their gut permeability increased, and serum IgG and cecal IgA increased significantly, indicating that the host immune activation was enhanced and induced changes in bacterial-specific humoral immunity. NF-κB is located in the downstream of the PI3K/Akt/mTOR signaling pathway, and mTOR mediates IKK-induced nuclear translocation of NF-κB to promote disease progression [230]. In LPS-stimulated Caco-2/HT29 co-cultures, IPA significantly inhibits the activation of the PI3K/AKT/mTOR signaling pathway and downregulates the expression of TNF-α, IL-8, and IL-6 inflammatory genes [221]. AHR is a ligand-dependent transcription factor, and numerous studies have shown that tryptophan derivatives serve as AHR ligands to stimulate the secretion of IL-22, thereby preventing chemically induced colitis [231,232]. Alexeev et al. reported that IPA activates AHR, reduces DSS-induced IFN-γ, TNF-α, and IL-1β, and reduces the severity of gut inflammation in mice [25]. We propose that IPA is an AHR ligand that plays a role in the resistance to gut inflammation through the IPA–AHR axis [53,233]. Although the complete biological pathway and regulated cytokines are still uncertain, IPA-related compounds show clear potential to be developed for treating patients with IBD.

At present, radiation therapy is a feasible and effective treatment for patients with cancer, but it is usually accompanied by some complications, resulting in bone marrow, hematopoietic system, and GI tract damage, clinically known as acute radiation syndrome (ARS) [234]. Researchers were surprised to find that oral IPA not only exhibited conventional gut-protective functions such as reversing radiation-induced colon shortening, as well as increasing gut villi and goblet cells [235]. Importantly, oral IPA also prevents the atrophy of hematopoietic organs (spleen and thymus), inhibits the loss of hematopoietic stem cells, and reduces the production of inflammatory and oxidative stress markers in the gut and peripheral blood. This series of protective mechanisms is mediated by the gut PXR/ACBP signaling and ultimately reduces mouse mortality. It is emphasized that the gavage of IPA alters the structure of irradiated gut microbiota, and IPA loses its protective effect in antibiotic-treated mice, indicating that gut microbiota play an irreplaceable role in the protective function of IPA. We speculate that some unidentified gut microbial products may also play a certain synergistic role.

The structure and function of the gut microbiota and its role in human health is a research hotspot. Combined with the pathological knowledge of gut dysbiosis and gut barrier permeability-mediated systemic diseases, targeting gut barrier homeostasis has emerged as an effective means of diagnosing and treating various diseases. The microbial metabolite IPA of dietary tryptophan, or IPA-producing probiotics, can be used as an adjuvant therapy for patients with gut disorders, and is expected to be a pharmaceutical component for diseases such as IBD, with good application prospects.

## 6. Discussion

Taken together, IPA produced only by the gut microbiota plays a direct or indirect role in various disease models that have not been previously recognized. Strikingly, IPA is well tolerated in current mouse models with no adverse effects [77,134]. However, there remain many problems and challenges in the clinical application of IPA, which need to be further explored in follow-up research.

Species diversity. Limited by difficulties in sample collection, numerous studies have focused on T84 monolayers [170], human cancer cell lines (HT-29, CACO-2) [221], and 3D spheroids [36], and most disease studies have used mouse models [94,225]. Therefore, the diversity of these pathways in human cells has been largely ignored. For example, after LPS stimulation, IPA promotes the secretion of IL-10 and decreases the production of TNF in murine bone marrow-derived macrophages (BMDM), which is consistent with the results of murine-derived colonic spheroids. However, IPA fails to show anti-inflammatory effects in the in vitro culture experiments of human peripheral blood mononuclear cells (PBMC) [36]. In vitro, IPA requires to be combined with indole to significantly activate the human PXR receptor, while IPA alone is sufficient to effectively activate mouse PXR [227]. Gesper et al. used rifampicin and 5-Pregnen-3β-ol-20-one-16α-carbonitrile (PCN) as human PXR and mouse PXR agonists, respectively. Chronic exposure to both IPA and rifampicin reduces the mitochondrial oxygen consumption rate (OCR) in Huh7 after FCCP co-incubation, but PCN does not alter OCR in murine HL-1, either alone or in combination with IPA [204]. These differential results highlight that the mouse model has certain differences from human physiology; thus, caution should be exercised in subsequent practical applications.

Determination of IPA dosage. It is well known that IPA can activate the PXR receptor to induce a variety of biological effects [13]. However, Wlodarska et al. showed that IPA does not significantly activate the PXR receptor, despite showing a trend of activation [36]. Cumulative studies have shown that IPA acts as a ligand or agonist of AHR and activates its target genes [25,52,53,178], while others have shown contradictory results [13,36]. We speculate that this may be related to the concentration, dosage, or duration of action. Moreover, the administration dose and response time of IPA are different. For example, IPA at a concentration of 1 mM but not 10 μM can modulate mitochondrial respiratory function when it is incubated with HL-1 for 24 h, and the therapeutic effect of IPA varies with exposure time [204]. Setting a gradient dosage for injection and continuously exploring the optimal therapeutic dosage are crucial for clinical application. Therefore, we systematically summarize the dosage and method of IPA in mouse models and cell lines (Table 3 and Supplementary Table S1) to facilitate subsequent experiments.

Role of gut microbiota/metabolites. Numerous studies tend to apply IPA to cells or mice alone, which largely ignores the role of gut microbiota. As described by Xiao et al., oral administration of IPA cannot inhibit ARS toxicity in antibiotic-treated mice, and the effectiveness of IPA must depend on the presence of microbiota [235]. Furthermore, IPA, despite being significantly positively correlated with ABI [205], fails to improve cardiometabolic profiles in WD-fed mice [21]. It is inferred that significant shifts in gut microbiota between groups induce phenotypic differences, or that undetermined remaining metabolites act synergistically with IPA. Wikoff et al. found that IPA could not be detected in the serum of mice when *C. sporogenes* colonized for less than 5 days [32]. Moreover, over time, after stopping treatment, the plasma IPA levels gradually decreased compared to those at the point of initial injection [134]. This prompts us to consider whether synergistic microbiota or metabolites are missed if the follow-up clinical laboratory fails to achieve results.

Administration of IPA. IPA is administered in vivo mainly by gavage, dissolving in sterile drinking water, and intraperitoneal injection (Table 3). As the absorption of the body varies according to the administration method [134], choosing the best method is crucial to the curative effect. Throughout all of the studies, most of the dissolution methods of IPA referred to the method described by Poeggeler et al. [71]. The reagents were mostly sourced from Sigma Aldrich except G-Clone Biotechnology Co., Ltd.

Organs targeted by IPA. IPA at a concentration of 10 μM enhanced the mitochondrial basal and maximal respiration in N2a cells stably transfected with APPsw (N2a-APPsw), while increasing mitochondrial membrane potential (MMP) and reducing the production of ROS [72]. However, IPA at a concentration of 1 mM but not 10 μM was able to modulate mitochondrial respiration after 24 h of incubation in HL-1, with prolonged treatment (24 h) reducing maximal respiration and short exposure (30 min) showing the opposite effect [204]. Inconsistently, IPA fails to alter mitochondrial basal respiration, MMP, and ROS production in murine HL-1. However, IPA reduces mitochondrial basal respiration in Huh7 and HUVECs, which may indicate that the sensitivity to IPA varies according to cell type and species origin. This leads to the conclusion that specific studies based on the species origin and cell type of the target organ are essential.

Insights for future research. Exploration of IPA-producing bacteria and determination of the IPA content in humans remain avenues to be explored in future studies. The identification of IPA-producing bacteria in in vitro culture has many constraints, and will be affected by the culture environment, such as low pH, which will interfere with the identification of target bacteria. Subsequently, the association between IPA content and gut microbiota should be analyzed with larger data to identify potential IPA-producing bacteria from the associated microbiota. The content of IPA in the human gut has not yet been reported, and the content in the serum is variable. An analysis of the IPA content in serum/feces in a large cohort is urgently needed to provide reference for IPA tolerance and injection volume in humans. Although IPA is still far from clinical application, the above research provides a new strategy for the prevention and treatment of complex diseases and drug development. Therefore, the above limitations should be overcome in future research to gradually explore the complex human mechanisms and improve the effective therapeutic results of IPA.

## 7. Conclusions

**Figure 3:**
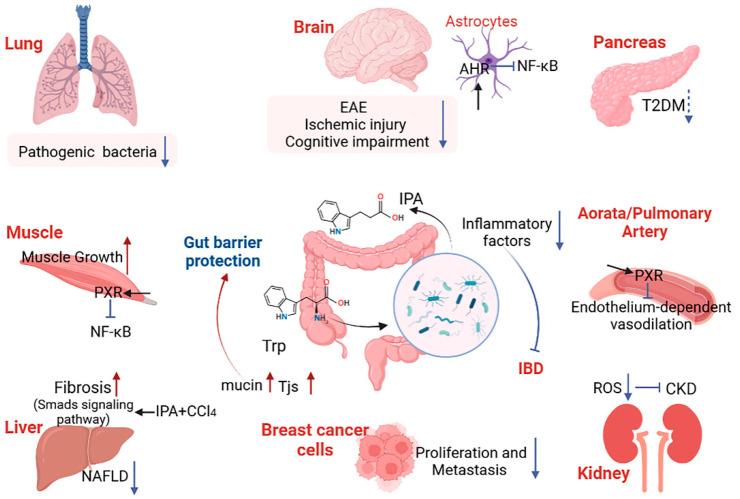
Summary of IPA production and functions. Red upward arrows indicate promotion, and blue downward arrows indicate inhibition. The dashed line indicates that further experimental verification is required. IPA: indole propionic acid, Trp: tryptophan, EAE: experimental autoimmune encephalomyelitis, T2DM: type 2 diabetes mellitus, NAFLD: Non-alcoholic fatty liver disease, CKD: chronic kidney disease, AHR: aryl hydrocarbon receptor, PXR: pregnane X receptor, ROS: reactive oxygen species, Tj: tight junction.

Dietary tryptophan ingested by the host is deaminated by gut microbiota to produce IPA, which targets various organs to perform biological functions through blood circulation (Figure 3). IPA stimulates goblet cells to secrete mucin and enhance the expression of TJs, thereby maintaining gut barrier homeostasis and attenuating the progression of inflammatory bowel disease. In addition, IPA is an effective hydroxyl radical scavenger without producing pro-oxidative intermediates, inhibiting ROS production and lipid peroxidation, reducing the expression of inflammatory factors, and ultimately maintaining the homeostasis of the body. As an excellent small-molecule metabolite, IPA can not only cross the blood–brain barrier and act on astrocytes to inhibit the NF-κB pathway, but it also protects nerve cells from oxidative or ischemic damage. For some complex metabolic diseases, such as CVD, NAFLD, and T2DM, IPA participates in disease treatment by stimulating corresponding receptors such as PXR or AHR through the specific gut–organ axis. The biological mechanism of IPA in vivo is complex. For example, although IPA effectively inhibits the activation of hepatic stellate cells and prevents liver fibrosis, it aggravates CCL4-induced liver fibrosis through the Smads signaling pathway in the presence of CCL4. In addition, the dose, treatment mode, action time, and even different target organs of IPA will affect the experimental effect. When translating disease treatment from laboratory research to clinical application, conclusions should be drawn with caution, taking into account the available evidence.

Further work is needed to explore the effects and mechanisms of IPA on host health and disease, especially in humans. However, it is undeniable that IPA has obvious therapeutic effects on many diseases, bringing additional benefits to animals and even humans, with the potential to improve the existing treatment program.

## Figures and Tables

**Table 1:** Gut bacterial species reported to produce IPA.

Producers	Phylum	Family	Genes Involved in the Production of IPA	References
*Clostridium sporogenes*	Firmicutes	Clostridiaceae	fldH, fldBC, acdA, etfA-etfB	[33,34,35]
*C.* *cylindrosporum*	Firmicutes	Clostridiaceae	-	[34]
*Peptostreptococcus asaccharolyticus*	Firmicutes	Peptoniphilaceae	-	[12]
*P. russellii*	Firmicutes	Peptostreptococcaceae	fldBC	[36]
*P. anaerobius*	Firmicutes	Peptostreptococcaceae	fldBC, acdA, etfA-etfB	[35,36]
*P. stomatis*	Firmicutes	Peptostreptococcaceae	fldBC	[36]
*C. botulinum*	Firmicutes	Clostridiaceae	fldH, fldBC, acdA, etfA-etfB	[33]
*C. caloritolerans*	Firmicutes	Clostridiaceae	-	[33]
*C. cadaveris*	Firmicutes	Clostridiaceae	fldBC, acdA, etfA-etfB	[35]

IPA: Indole propionic acid. fldH: indolelactate dehydrogenase gene, fldBC: indolelactate dehydratase gene cluster, acdA: acyl-CoA dehydrogenase gene, etfA-etfB: electron transport factor genes. “-” indicates that it was obtained by isolation and culture in vitro without genome information.

**Table 2:** Bacteria and fungi sensitive to IPA.

Species	Phylum	Family	References
*Mycobacterium avium*	Actinobacteria	Mycobacteriaceae	[133,134]
*Mycobacterium kansasii*	Actinobacteria	Mycobacteriaceae	[133]
*Mycobacterium chelonae*	Actinobacteria	Mycobacteriaceae	[133]
*Mycobacterium tuberculosis*	Actinobacteria	Mycobacteriaceae	[133,134]
*Mycobacterium fortuitum*	Actinobacteria	Mycobacteriaceae	[133]
*Mycobacterium abscessus*	Actinobacteria	Mycobacteriaceae	[133]
*Mycobacterium smegmatis*	Actinobacteria	Mycobacteriaceae	[133,134]
*Mycobacterium bovis BCG*	Actinobacteria	Mycobacteriaceae	[133,134]
*Candida albicans*	Ascomycota	Debaryomycetaceae	[135]
*Candida parapsilosis*	Ascomycota	Debaryomycetaceae	[135]
*Trichosporon beigelii*	Basidiomycota	Trichosporonaceae	[135]
*Malassezia furfur*	Basidiomycota	Malasseziaceae	[135]
*Trichophyton rubrum*	Ascomycota	Arthrodermataceae	[135]
*Aspergillus flavus*	Ascomycota	Aspergillaceae	[135]
*Saccharomyces cerevisiae*	Ascomycota	Saccharomycetaceae	[135]
*Legionella pneumophila*	Proteobacteria	Legionellaceae	[136]

IPA: Indole propionic acid.

**Table 3:** Dosage and administration time of IPA in mouse models.

Subjects	Age	Dosage	Administration Mode	Dosing Time	Reference
C57BL/6J mice	4–5 months	0.1 mg/mL	Drinking water	5 months	[21]
C57BL/6 mice	8–10 weeks	0.1 mg/mL	Drinking water	9 days	[25]
C57BL/6 mice	6–8 weeks	10.0, 20.0, 40.0 mg/kg	Oral gavage	4 days	[227]
SW/SWGF mice	7–8 weeks	20.0 mg/kg	Oral gavage	4 days	[227]
C57BL/6 mice	8–10 weeks	20.0 mg/kg	Oral gavage	15 days	[52]
C57BL/6 mice	5–6 weeks (20–22 g)	200.0 mg/L	Drinking water	2 weeks	[216]
C57BL/6J mice	6–8 weeks	7.5 mg/mL × 0.2 mL/mice	Oral gavage	15 days	[235]
BALB/c athymic nude mice	4 weeks	7.5 mg/mL × 0.2 mL/mice	Oral gavage	4 days	[235]
SD rats	6 weeks + 8 weeks (Dietary induction)	20.0 mg/kg	Oral gavage	8 weeks	[29]
ICR mice	Male (28–32 g) Female (25–30 g)	100.0 mg/kg	Oral gavage	8 weeks	[94]
Wistar rats	Weight approximately 160 g	12.0 mg/kg	Intraperitoneal injections	10 days (twice daily)	[121]
C57BL/6	6–8 weeks (weighing approximately 25 g) + 1 week (adapt to the environment)	20.0 mg/kg	Oral gavage	8 weeks	[95]
Mongolian gerbils (*Meriones unguiculatus*)	6 months (BW 65–75 g)	10.0 mg/kg	Oral gavage	15 days	[75]
SD rats	14 weeks	30.0 mg/kg	Intraperitoneal injections	1 week	[27]
SD rats	180–200 g	20.0 mg/kg	Intraperitoneal injections	4 h	[71]
SD rats	180–200 g	1 μL (20.0 nmol in 0.1 M PBS)	Injected unilaterally into the striatum	1 h	[71]
DIO mice	23–26 weeks (maintained on HFD for 19–22 weeks)	20.0 mg/kg	Oral gavage	4 days	[170]
BALB/c mice	3 months	0.2 mg/kg	Oral gavage	14 days	[30]
BALB/c mice	8–10 weeks + 14 days (TB induced)	100.0 mg/kg	Oral gavage	4 weeks (6 days/week)	[134]
Wistar rats	Weight approximately 160 g	12.0 mg/kg	Intraperitoneal injections	10 days (twice daily)	[122]
SWGF mice	6–8 weeks	10.0, 20.0, 40.0 mg/kg	Intraperitoneal injections	6 h	[32]

DIO mice: Diet-induced obese C57BL/6 mice (maintained on HFD for 19–22 weeks), SD rats: Sprague–Dawley rats, SWGF mice: Swiss Webster Germ-Free mice.

## Data Availability

Not applicable.

## Supplementary Materials

The following supporting information can be downloaded at: https://www.mdpi.com/article/10.3390/nu15010151/s1, Table S1. Dosage, co-culture time, and results of IPA in cell lines.

## Abbreviations

AD	Alzheimer’s disease
AHR	aryl hydrocarbon receptor
CKD	chronic kidney disease
CNS	central nervous system
CVD	cardiovascular disease
CYPs	Cytochrome P450 enzymes
eNOS	endothelial nitric oxide synthase
GI	gastrointestinal
GLP-1	glucagon-like peptide-1
HCC	hepatocellular carcinoma
HK-2	proximal tubular cells
HL-1	murine cardiomyocytes
Huh7	human hepatoma cell line
IAA	indole acetic acid
IBD	inflammatory bowel disease
IPA	indole propionic acid
LPS	lipopolysaccharide
LX-2	human hepatic stellate cell line
MDA	malondialdehyde
MMP	mitochondrial membrane potential
MUC2	mucin 2
NAFLD	Non-alcoholic fatty liver disease
NASH	non-alcoholic steatohepatitis
OAT	organic anion transporter
PPARs	Peroxisome proliferator-activated receptors
PXR	pregnane X receptor
ROS	reactive oxygen species
SD	Sprague–Dawley
Stat3	transcription 3
TB	tuberculosis
TGF-β1	transforming growth factor-β1
TNF-α	tumor necrosis factor-α
Trp	tryptophan
TJ	tight junction
T2DM	Type 2 diabetes mellitus
WD	Western diet
