# Supplementary material for: Extensive Summary of the Important Roles of Indole Propionic Acid, a Gut Microbial Metabolite in Host Health and Disease
Source: Nutrients. 2022 Dec 28;15(1):151. doi: 10.3390/nu15010151 (PMC9824871; doi:10.3390/nu15010151)
Supplement: Supplementary file 1 [file nutrients-15-00151-s001.zip › nutrients-2107264-Table S1.pdf]

Supplementary Table S1. Dosage, co-culture time, and results of IPA in cell lines

Cell line	IPA concentration	Co-incubation time	Result	Reference
N2a-APPsw	10 μM	48 h	IPA increases basal and maximal respiratory rates, increases MMP, and inhibits ROS production	[72]
N2a	10 μM	48 h	IPA increases the maximum respiratory rate of N2a cells, increases MMP, and inhibits ROS production	[72]
HL-1	1 mM, 10 μM	24 h, 6 h, 2 h, 30 min or direct injection	After 24 h incubation, 1 mM IPA decreases maximal respiration and spare capacity; 1 mM, 10 μM IPA increases maximal respiration and spare capacity at 30 min of incubation	[204]
Huh7	1 mM	24 h	Both maximal respiration and basal respiration are reduced	[204]
HUVEC	1 mM	24 h	Basal respiration decreases significantly, and the maximum respiration tends to decrease	[204]
Microsomes were pre-incubated with IPA and then incubated for 20 min in the presence of $FeCl_3$ (0.2 mM), ADP (1.7 mM), and NADPH (0.2 mM)	10, 3, 2, 1, 0.3, 0.1, 0.01, or 0.001 mM	30 min	Only high physiological concentration of IPA can inhibit Fe^{3+}-induced lipid peroxidation	[100]
Calf thymus DNA (500 μg) treated with $CrCl_3$ (0.5 mM) plus H_2O_2 (0.5 mM)	0.25, 0.5, 1, 2.5, 5, or 10 μM	1 h	IPA dose-dependently inhibits Cr (III) plus H_2O_2-induced formation of 8-OH-dG in DNA	[101]
LX-2 with or without TGF-β1 (5 ng/mL) treatment	100 μM	24 h or 48 h	IPA inhibits cell adhesion, migration and fibrosis in LX-2 cells	[99]
Primary aorta-derived endothelial cells isolated from $Pxr^{+/+}$ and $Pxr^{-/-}$ mice	1 μM	24 h	IPA activates PXR, reduces endothelial eNOS expression, and normalizes abnormal vasodilatory responses	[216]
Aortic rings were cultured for 24 h with or without IPA	0.1 μM	24 h	IPA can directly affect endothelium-dependent vascular responses in isolated aortic rings	[216]
Colonic intestinal epithelial T84 cells with IPA alone or in the presence of IFN-γ and TNF-α	0.5, 5, 100 μmol/L	24 h	IPA dose-dependently reduces cytokine-induced permeability without altering cellular permeability under basal conditions	[170]

Human T84 cells intestinal epithelial cells	0.01, 0.1, 1 mmol/L	6 h	IPA concentration-dependently induces the transcription of IL-10R1	[25]
HIOs	1 mmol/L	Over 24 h	IPA promotes the transcription of IL-10R1 in HIOs and reaches statistical significance at 24 h	[25]
HK-2 were pretreated with or without IPA and then incubated with IS (250 μmol/L) for the indicated times	1000 μmol/L	30 min	IPA inhibits IS-induced expression of AHR, CYP1A1, TGF-β1, MCP-1, and expression and phosphorylation of Stat3	[117]
Caco-2/HT29 coculture or HT29 cells treated with IPA with or without LPS (1 μg/mL)	0.1 mM	24 h	IPA increases goblet cell secretory products (TFF3 and RELMβ), promotes the expression of mucins (MUC2, MUC4) and tight junctions (claudin-1, occludin, and ZO-1), and inhibits LPS-induced inflammatory factors (TNF-α, IL-8 and IL-6)	[221]
C2C12 cells were pretreated with IPA for 48 h, and then treated with 100 ng/mL and 1000 ng/mL LPS for 12 h and 24 h, respectively	0.1 mM, 0.25 mM, 0.5 mM	48 h	IPA alleviates LPS-induced cellular inflammatory response by activating PXR in muscle cells, inhibiting NF-κB signaling pathway, and regulating the transcription of miRNAs	[156]
C2C12 cells	0.1, 0.25, 0.5 mM	24 h	0.1 mM IPA significantly increases myoblast viability	[156]
MODE-K cells were irradiated with 6 Gy, with or without IPA supplementation	9.45, 14.175, 18.9, 37.8, 56.7, 75.6 μg/mL	72 h	IPA dose-dependently promotes the proliferation of irradiated MODE-K cells	[235]
HIEC-6 cells were irradiated with 6 Gy, with or without IPA supplementation	18.9, 28.35, 33.075, 37.8, 56.7, 75.6 μg/mL	72 h	IPA dose-dependently promotes the proliferation of irradiated HIEC-6 cells	[235]
ACBP siRNA-treated HIEC-6 cells were irradiated with 4 Gy radiation with or without IPA	37.8 μg/mL	72 h	Depletion of ACBP prevents the protective effect of IPA against radiation in HIEC-6 cells	[235]
Homogenates of porcine thyroid glands were incubated with IPA with or without $KBrO_3$ (5 mM)	0.01, 0.1, 0.5, 1.0, 5.0, 7.5, 10 mM	30 min	IPA fails to affect basal lipid peroxidation and to inhibit $KBrO_3$-induced elevation of MDA + 4-HDA levels. The lack of protective effect of potential antioxidants under in vitro	[122]

			conditions does not preclude their ability in living organisms	
Striatal tissue homogenate was incubated with a final concentration of 5 mM H_2O_2 with or without IPA	2 μM–2 mM	60 min	IPA concentration-dependently attenuates H_2O_2-induced MDA formation	[71]
4T1 murine breast cancer cells	0.1, 0.2, 0.4, 0.8 μM	24 h, 7 days	IPA reduces the proportion of cancer stem cells by inhibiting epithelial-to-mesenchymal transition, inducing oxidative stress and cellular energy stress	[30]
SKBR-3 human breast cancer cells	0.1, 0.2, 0.4, 0.8 μM	24 h	Inhibition of IPA on the proliferation of breast cancer cells is not specific to 4T1 cells	[30]
Human primary fibroblasts cells	0.1, 0.2, 0.4, 0.8 μM	24 h	IPA is not cytotoxic to primary, untransformed human fibroblasts and does not inhibit cell proliferation	[30]
4T1, CH223191 (AHRi), Ketoconazole (PXRi)	0.4, 0.8 μM	24 h	AHR and PXR are responsible for IPA-induced antitumor effects	[30]
Primary hippocampal neurons were differentiated for 7–10 days and then treated with 1 μM Aβ-(1-42) with or without IPA	1 μM	24 h	The addition of IPA prevents death of primary neurons exposed to Aβ	[68]
SK-N-SH human neuroblastoma cells were exposed to 1 mM DDTC or 50 μM H_2O_2 with or without IPA	1, 10, 50, 100 μM	24 h	IPA prevents DDTC- or H_2O_2-mediated neuroblastoma cell death	[68]
HepG2 cell lines supplemented with 100 μM oleic acid were treated with different concentrations of IPA or 10 μM simvastatin	10, 25, 50 μM	Overnight	IPA dose-dependently inhibits oleic acid-induced lipid accumulation and reduces transcription of key genes for lipogenesis	[94]
HKCI-2/HKCI-10 (NASH–HCC cell lines) treated with cholesterol (200 μg/mL)	10, 100 μM	96 h	IPA inhibits cholesterol-induced triglyceride accumulation and cell proliferation in HKCI-2 and HKCI-10	[86]
Murine J774A.1 macrophages were pretreated with IPA and	250 μM, 500 μM	1 h	IPA inhibits LPS-induced p65 phosphorylation and reduces the expression of pro-inflammatory cytokines in a dose-dependent manner	[29]

then treated with 500 ng/mL LPS for 30 min				
Candida albicans in the presence or absence of cyclosporin A (1 μg/mL) and ruthenium red (1.5 μg/mL)	5 μg/ml	4 h	Under the mediation of Ca^{2+}, IPA induces apoptosis in *Candida albicans* by regulating cellular respiration, depolarizing mitochondrial membrane potential, and triggering DNA fragmentation	[135]
Protoplasts from *Candida albicans* in the presence or absence of cyclosporin A (1 μg/mL) and ruthenium red (1.5 μg/mL)	5 μg/ml	2 h		[135]
Legionella pneumophila in the presence and absence of Trp (vigorous shaking)	0.53, 2.64, 5.28, 26.43 μM	48 h	*Legionella pneumophila* cultured under vigorous shaking conditions is more sensitive to IPA compared to that cultured under static conditions. Trp prevents the antibacterial function of IPA, and the effect of IPA is time-dependent	[136]
Legionella pneumophila in the presence and absence of Trp (static culture)	5.28, 26.43, 52.85, 105.70 μM	96 h		[136]
Human monocytes were infected with *L. pneumophila* for 1 h before adding IPA (in a humidified 5% CO_2 incubator)	5.28, 26.43, 105.70, 211.40 μM	96 h	The susceptibility of *Legionella pneumophila* grown in human monocytes to IPA was significantly lower than that grown in culture medium. The effect of IPA is time-dependent	[136]
293T cells were treated with IPA with or without 1 mM indole	1, 10, 100, 1000 μM	48 h	The combination of IPA and indole significantly activates human PXR (hPXR), and mouse PXR (mPXR) is effectively activated by IPA alone in vitro, and fails to prove that IPA activates AHR	[227]
Nr1i2 $^{+/+}$, hNr1i2 and Nr1i2 $^{-/-}$ mouse jejunal chorionic enterocytes were treated with OLA (5 mg/mL), indole (1 mM) and IPA	1 μM	3 h	PXR is an enterocyte target for IPA	[227]
BMDMs were treated with IPA, followed by treatment with rmIL-4 (PeproTech) for 24 h, and then stimulated with 20 ng/mL LPS for 18 h.	100 μM	6 h	IPA significantly increases IL-10 production and decreases TNF production	[36]

Spheroids	100 μM	72 h	IPA fails to significantly induce the expression of PXR target genes Ugt1a1 and Mdr-1	[36]
Co-cultures of colonic spheroids and BMDMs were pre-treated with IPA and then stimulated with 20 ng/mL LPS for 24 h	100 μM	48 h	IPA inhibits IL-6 and TNF secretion and stimulates IL-10 secretion in co-cultures	[36]

BMDMs: Bone marrow-derived macrophages, DDTC: diethyldithiocarbamate (an inhibitor of superoxide dismutase-1), HIOs: Human intestinal organoids, HK-2: Serum-starved proximal tubular cells, HL-1: Murine cardiomyocytes, Huh7: Human hepatoma cell line, HUVEC: Human umbilical vein endothelial cells, IL-10R1: IL-10 receptor ligand-binding subunit, LX-2: Human hepatic stellate cell line, N2a-APPsw: N2a cells stably transfected with APPsw, OLA: oxindolyl-L-alanine, 8-OH-dG: 8-hydroxydeoxyguanosine.
